# Autonomic dysfunction in progressive supranuclear palsy

**DOI:** 10.1007/s00415-022-11347-w

**Published:** 2022-08-30

**Authors:** Francesca Baschieri, Maria Vitiello, Pietro Cortelli, Giovanna Calandra-Buonaura, Francesca Morgante

**Affiliations:** 1 Dipartimento di Scienze Biomediche e Neuromotorie, Università di Bologna, Bologna, Italy; 2 Neurology Unit, “M. Bufalini” Hospital, AUSL Romagna, Cesena, Italy; 3 IRCCS Istituto delle Scienze Neurologiche di Bologna, Bologna, Italy; 4 Neurosciences Research Centre, Molecular and Clinical Sciences Research Institute, St George’s University of London, London, UK; 5 Department of Experimental and Clinical Medicine, University of Messina, Messina, Italy

**Keywords:** Progressive supranuclear palsy, Autonomic dysfunction, Orthostatic hypotension, Urinary incontinence, Photophobia

## Abstract

**Background:**

The degree of involvement of the autonomic nervous system in progressive supranuclear palsy (PSP) has been investigated in several studies, often providing conflicting results. There is a need for a better characterization of autonomic dysfunction in PSP, to enhance our understanding of this highly disabling neurodegenerative disease including patients’ needs and possibly be of value for clinicians in the differential diagnosis among Parkinsonian syndromes.

**Methods:**

We applied a systematic methodology to review existing literature on Pubmed regarding autonomic nervous system involvement in PSP.

**Results:**

PSP reported quite frequently symptoms suggestive of autonomic dysfunction in all domains. Cardiovascular autonomic testing showed in some cases a certain degree of impairment (never severe). There was some evidence suggesting bladder dysfunction particularly in the storage phase. Dysphagia and constipation were the most common gastrointestinal symptoms. Instrumental tests seemed to confirm sudomotor and pupillomotor disturbances.

**Conclusions:**

PSP patients frequently reported visceral symptoms, however objective testing showed that not always these reflected actual autonomic impairment. Further studies are needed to better delineate autonomic profile and its prognostic role in PSP.

## Introduction

Progressive supranuclear palsy (PSP) is a sporadic tauopathy, characterized by accumulation of tau isoform with four repeat sequences (4R-tauopathy) in several areas of the central nervous system. Neuropathological features include neurofibrillary tangles, neuropil threads, tufted astrocytes, oligodendroglial coiled bodies, neuronal loss and gliosis particularly in subcortical structures (i.e. basal ganglia, subthalamic nucleus and brainstem), and later in cortical areas (mainly frontal and parietal) and cerebellar structures [1].

The main clinical phenotype, known as Richardson’s syndrome, is characterized by vertical supranuclear gaze palsy and postural instability with early falls. This is one among several other possible clinical manifestations of PSP pathology [2] which led to the latest clinical diagnostic criteria [3].

Predominant, otherwise unexplained autonomic failure is listed among the mandatory exclusion criteria of PSP, e.g. orthostatic hypotension (OH) after 3 min standing ≥ 30 mmHg systolic or ≥ 10 mmHg diastolic suggestive of multiple system atrophy (MSA) or Lewy body disease (LBD) [3]. Severe autonomic failure is indeed a prominent and well-studied feature of alpha-synucleinopathies. Nevertheless, several studies based on autopsy-proven PSP showed that autonomic features might be present also in PSP. Therefore, there is a need for better characterization of autonomic nervous system involvement in PSP. This may help to enhance our understanding of this highly disabling neurodegenerative disease including patients’ needs and possibly be of value for clinicians in the differential diagnosis among parkinsonian syndromes. To this aim, we reviewed the existing literature regarding autonomic nervous system involvement in PSP.

## Methods

In this review, we applied a systematic methodology to literature search on PubMed for articles in English language published up to May 2022. We used the following combination of MeSH terms: “progressive supranuclear palsy” and “autonomic nervous system”, “autonomic testing”, “cardiovascular system”, “orthostatic hypotension”, “hypertension”, “supine hypertension”, “blood pressure”, “urinary system”, “urinary incontinence”, “urinary urgency”, “urinary retention”, “erectile dysfunction”, “gastrointestinal system”, “dysphagia”, “constipation”, “pupillomotor system”, “pupils”, “photophobia”, “sudomotor system”, “sweating”, “thermoregulation”, “vasomotor system”, “cold hands”, “secretomotor system”, “lacrimation”, “salivation”, “non motor symptoms”.

We included studies which enrolled patients with clinical or pathologically confirmed diagnosis. Only articles published in peer-reviewed journals in English were included. Given the paucity of studies on this topic, all study designs including case reports were accepted. We created a database of primary search results and then we compiled a list of non-duplicate studies according to inclusion and exclusion criteria. Relevant studies from the reference list of primary search results were identified and included in the review process. Papers were considered relevant when they aimed at investigating autonomic nervous system symptoms and function in PSP according to appropriate procedures for this purpose. These included validated questionnaires and scales, standardized interview, review of medical records, objective testing.

## Results

### Cardiovascular system

The cardinal sign of failure of cardiovascular autonomic control is neurogenic OH (NOH). OH is defined as a sustained reduction of at least 20 mmHg of systolic blood pressure (SBP) and/or 10 mmHg of diastolic blood pressure (DBP) within 3 min of standing or head-up tilt test (HUTT) [4]. Common symptoms may be related to cerebral hypoperfusion (dizziness; visual disturbances like blurred or tunnel vision, scotoma, greying out, blacking out, colour defects; syncope and cognitive slowing), muscular hypoperfusion (suboccipital/paracervical “coat-hanger” and low back pain) or be less specific like weakness, lethargy and fatigue. These symptoms occur on standing and typically subside by lying down [5].

OH can be neurogenic or it can be secondary to other causes (non-NOH), including drugs that lower blood pressure.

Previous studies addressed OH by evaluating symptoms with questionnaires and/or by measuring orthostatic blood pressure drop during standing test or HUTT. However, OH may also be completely asymptomatic and detected only by measuring blood pressure. Conversely, some of the symptoms are aspecific and may not correspond to an objective drop in blood pressure. Hence, the correct diagnosis of OH requires objective evaluation while recording symptoms is useful to assess its clinical impact [6].

Orthostatic symptoms investigated by means of questionnaires (Non-Motor Symptom Scale (NMSS), Autonomic Symptom Questionnaire) were reported by 20–50% of PSP patients [7–10] and by 13% on structured interview [11]. One study reported a mean score of 12 for the cardiovascular domain on the SCOPA-Aut questionnaire, which was intermediate between the score of MSA (the highest) and Parkinson’s disease (PD) (lowest) [12]. These studies did not have an objective confirmation of OH, nevertheless the lower prevalence of OH reported by studies with objective testing (described later) seems to suggest that the questionnaires may overestimate OH. Indeed, Bae et al. found that orthostatic symptoms at the Autonomic Dysfunction Questionnaire were complained by 42% of PSP patients but only 33% actually presented OH and no correlation was found between symptoms and OH objective presence [13]. Likewise, 63% of PSP patients reported cardiovascular symptoms during a standardized clinical interview, yet autonomic testing disclosed OH only in 16% of cases [14]. Similarly, even though 52% of PSP patients reported light-headedness on standing, none had OH on standing [15].

The prevalence of OH assessed objectively with instrumental tests in PSP ranged from 0 to 45% [13–24]. The large variability across studies could be ascribed to different study designs and methods of assessments. Nine studies were cross sectional [13–15, 17–22] and 3 retrospective with pathology confirmation [16, 23, 24]. HUTT was performed in 6 studies [14, 19–23], standing in 4 [15–17, 24] and both HUTT and standing in further 2 studies [13, 18]. The criteria for OH were mostly a − 20 mmHg sustained drop in SBP on standing/HUTT except for one study [19] that used a − 30 mmHg cut-off. The list of studies assessing cardiovascular autonomic function is reported in Table 1.

**Table 1:** Studies assessing OH, cardiovascular autonomic function and related symptoms in PSP

References	Study design	N° PSP (m/f)	Age (y)	Disease duration (y)	Assessment	OH (%)	Sympathetic function	Parasympathetic function	Symptoms (%)
Van Dijk et al. [39]	Cross-sectional	11 (6/5)	75.5 ± 6.4	3.5 ± 2.1	Standing, VM, DB, isometric exercise	n/a	P	P	n/a
Sandroni et al. [40]	Retrospective	32 (19/13)	65.6 ± 6.6 (at onset)	n/a	HUTT, VM, DB	n/a	P	P	n/a
Gutrecht [41]	Cross-sectional	9 (4/5)	71 [66–84]	3.6 [2–6]	Standing, VM, DB, isometric exercise, cold pressor	n/a	P	P	n/a
Litvan et al. [25]	Retrospective + pathology	24	63 ± 2 (onset)	78 ± 8 (months)	Review of medical records	Not predictive of PSP	n/a	n/a	n/a
Yoshita [47]	Cross-sectional	14 (8/6)	68 [62–74]	5 [1–11]	MIBG	n/a	N	n/a	n/a
Wenning et al. [16]	Retrospective + Pathology	24 (15/9)	63.1 ± 6.9 (onset)	79 ± 44 (months)	Standing, review of medical records	45%	n/a	n/a	n/a
Kimber et al. [18]	Cross-sectional	35 (24/11)	65.5 ± 1.3	3.8 ± 0.6	Standing, HUTT, VM, DB, isometric exercise, cutaneous cold, mental arithmetic, clonidine challenge	0	N	N	n/a
Brefel-Courbon et al. [44]	Cross-sectional	8 (4/4)	71 ± 1	4 ± 1	HUTT, BPV, HRV	n/a	N	N	n/a
Holmberg et al. [45]	Cross-sectional	15 (9/6)	68.7 ± 4.5	4.7 ± 2.6	HUTT, DB, HRV	n/a	N	N	n/a
Deguchi et al. [46]	Cross-sectional	11 (7/4)	65 ± 6	3.6 ± 2.2	HUTT, VM, DB, noradrenaline plasma levels	n/a	N	N	n/a
Kikkawa et al. [19]	Cross-sectional	12 (9/3)	70.4 ± 6.3	3.7 ± 2.8	HUTT, HRV, noradrenaline plasma levels	0	N	N	n/a
Nagayama et al. [48]	Longitudinal	7	n/a	n/a	MIBG	n/a	N	n/a	n/a
Friedrich et al. [42]	Cross-sectional	32 (22/10)	66 ± 7	3.5	HUTT, VM, DB, HRV, BPV, baroreflex sensitivity	n/a	P	P	n/a
Schmidt et al. [14]	Cross-sectional	32 (22/10)	66 ± 7	3.5	HUTT, VM, DB, standardized clinical interview	16%	P	P	63
Bae et al. [13]	Cross-sectional	21 (11/10)	69.4 ± 6.9	3.5 ± 2.1	Standing or HUTT, autonomic dysfunction questionnaire	33%	N	n/a	42
Schmidt et al. [20]	Cross-sectional	32 (22/10)	66 ± 7	3.5	HUTT, VM	16%	P	P	n/a
Schmidt et al. [21]	Cross-sectional	25 (18/7)	65 ± 7	3.5	HUTT, VM, DB, 24-h ABPM	8%	P	P	n/a
Reimann et al. [22]	Cross-sectional	32 (22/10)	65.8 ± 6.9	3.5	HUTT, VM, DB, 24-h ABPM, autonomic dysfunction score (interview)	16%	P	P	63
Friedrich et al. [43]	Cross-sectional	32 (22/10)	66 ± 7	3.5	HUTT, VM, DB, baroreflex sensitivity	n/a	P	P	n/a
Colosimo et al. [11]	Longitudinal	30 (16/14)	69.7 ± 6.3	3.5 ± 3.1	Interview	n/a	n/a	n/a	13.3
Tateno et al. [58]	Cross-sectional	9	n/a	n/a	MIBG	n/a	N	n/a	n/a
Kurata et al. [50]	Retrospective	77 (47/30)	72.6 ± 6.0	5.7 ± 3.9	MIBG	n/a	N	n/a	n/a
Berganzo et al. [12]	Cross-sectional	20 (11/9)	68.3 ± 7.2	5.0 ± 2.4	SCOPA-Aut	n/a	n/a	n/a	12.28 (mean score)
Respondek et al. [26]	Retrospective + pathology	100 (45/55)	65.2 ± 0.9 (onset)	8.7 ± 0.4	Review of medical records	Not predictive of PSP	n/a	n/a	n/a
Koga et al. [28]	Retrospective + pathology	15 (9/6)	66 ± 11 (onset)	8.1 ± 3	Review of medical records	14	n/a	n/a	21
Ou et al. [9]	Case–control	27 (16/11)	65.1 ± 8.4	3.6 ± 2.1	NMSS	n/a	n/a	n/a	18.5
Radicati et al. [7]	Cross-sectional	50 (22/28)	69.8 ± 9.0	3.8 ± 2.1	NMSS	n/a	n/a	n/a	50
Respondek et al. [27]	Retrospective + pathology	206	66.2 ± 0.6 (onset)	7.9 ± 0.3	Review of medical records	Not predictive of PSP	n/a	n/a	n/a
Nojszewska et al. [8]	Cross-sectional	37	67.5 ± 6.1	4.6 ± 3.6	HRV supine and at DB, modified Autonomic Symptom Questionnaire	n/a	n/a	P	50
Van Gerpen et al. [23]	Retrospective + Pathology	14 (7/7)	68 [40–77] (onset), 74 [54–91] (death)	8 [4–15]	HUTT, VM, DB, CASS	0	N	P	n/a
Oliveira et al. [24]	Retrospective + Pathology	103 (64/39)	66.8 [62.1–72.5] (onset), 74.6 [70.4–81.4] (death)	n/a	Standing, review of medical records	9%	n/a	n/a	n/a
Miki et al. [29]	Retrospective + pathology	13 (9/4)	68.5 ± 6.6 (onset)	7.7 ± 3.7	Review of medical records	15	n/a	n/a	n/a
Kamada et al. [52]	Retrospective	6 (2/4)	74.1	4.0	MIBG	n/a	P	n/a	n/a
Schubert et al. [51]	Cross-sectional	25 (13/12)	69 ± 7	5.6 ± 2.7	MIBG	n/a	N	n/a	n/a
Chaithra et al. [10]	Cross-sectional	76 (53/23)	62.04 ± 7.10	2.68 ± 2.10	NMSS	n/a	n/a	n/a	27.6
Dubbioso et al. [15]	Cross-sectional	27 (18/9)	70 ± 7.4	3.6 ± 2.1	Standing SCOPA-Aut SFN-SIQ	0	n/a	n/a	52% 30%
Liu et al. [17]	Cross-sectional	31 (18/13)	70.5 ± 8.3	4.0 ± 2.7	Standing 24-h ABPM	0	n/a	n/a	n/a

*BPV* blood pressure variability, *CASS* Composite Autonomic Severity Score, *DB* deep breathing, *HRV* heart rate variability, *HUTT* head-up tilt test, *MIBG* meta-iodo-benzylguanidine myocardial scintigraphy, *N* normal, *NMSS* Non-Motor Symptom Scale, *OH* orthostatic hypotension, *P* pathological (vs controls), *SCOPA-Aut* Scales for Outcomes in Parkinson’s Disease-Autonomic, *SFN-SIQ* small fiber neuropathy symptoms inventory questionnaire, *VM* Valsalva manoeuvre, *24-h ABPM* 24 h ambulatory blood pressure monitoring. Age and disease duration are reported as mean±  standard deviation or [range]

OH defined as a sustained drop of at least 20 mmHg systolic blood pressure and/or at least 10 mmHg diastolic blood pressure within 3 min of standing or HUTT

Among cross-sectional clinical studies, 4 did not find OH in any PSP patient [15, 17–19], whereas the rest found a prevalence between 8 and 33% [13, 14, 20–22]. However, 4 out of 5 of those who found some degree of OH were conducted on the same cohort of PSP [14, 20–22]. Retrospective studies with pathological confirmation also provided conflicting results. Van Gerpen et al. did not find OH in 14 PSP previously assessed with formal autonomic testing [23]. Oliveira and colleagues found instead a 9% prevalence of documented or symptomatic OH in their large cohort of PSP (104 patients, the study with the largest numerosity) [24]. Conversely, Wenning and co-workers reported the highest prevalence (45%) of OH tested with standing at bedside in 24 patients [16].

The potential, confounding, hypotensive role of dopaminergic medication should be taken into account when interpreting these results. In 3 studies, cardiovascular autonomic tests were performed after a washout [18, 22, 23]. In the remaining, these medications were not discontinued prior to testing [14, 20, 21] or it was not specified [13, 15–17, 19, 24]. It is noteworthy that 2 out of 3 studies where dopaminergic drugs were discontinued prior to HUTT found no OH. This may imply that the higher prevalence found in other studies may at least partly reflect OH secondary to medications.

Some studies highlighted the discrepancy between the number of patients with OH according to criteria and those who were also symptomatic, the latter being usually a minority. For example, Schmidt et al. found that while 16% had OH, only 6% were symptomatic during HUTT [14]. Similarly, Bae and collaborators found no correlation between complaints of orthostatic intolerance and the presence of OH [13].

A similar median latency for OH (30 months [16] and 2 years [24]) was reported by some retrospective studies with regular follow-up.

Other retrospective studies with pathological confirmation based on review of medical records concluded that OH is not a predictive feature of PSP pathology, but the precise number of patients with OH was not reported [25–27]. OH was recorded in medical charts of 2 out of 14 patients with a pathological PSP diagnosis that had previously received a clinical diagnosis of MSA and indeed OH was significantly more frequent in MSA [28]. Another study with a similar design found a very much alike prevalence (15%) of severe OH (− 30/− 15 mmHg) in PSP [29]. These last two studies explored a selected population of patients (pathologically proven PSP that were misdiagnosed in life as MSA) therefore prevalence values are relative, however they show how even in this context OH is an uncommon finding.

One of the main issues that arise when dealing with OH is to identify the cause. Heart rate (HR) response to the orthostatic test has been proven useful to differentiate between NOH and non-NOH. A panel of experts recommended that in the presence of OH, a ∆HR < 15 on standing suggests NOH (i.e. a blunted reflex increase) [30]. Subsequently it was suggested that the ratio between ∆HR and the drop in SBP at the 3rd min of HUTT (∆HR/∆SBP) < 0.49 bpm/mmHg could identify NOH with better sensitivity and specificity [31, 32]. The gold standard to detect the neurogenic nature of OH still remains the Valsalva manoeuvre, which needs to be performed in a specialized laboratory under continuous blood pressure monitoring and hence it is not widely available.

NOH was specifically addressed only by Van Gerpen and colleagues [23], who reviewed medical charts and autonomic testing of 14 autopsy-confirmed PSP patients. NOH, defined by a SBP drop of at least − 30 mmHg at 5 min of HUTT associated with a pathological Valsalva manoeuvre or a ∆HR/∆SBP < 0.5 or Composite Autonomic Severity Score (CASS) adrenergic subscore > 2, was not found in PSP.

About half the patients with NOH presents supine hypertension (SH), defined by a SBP ≥ 140 mmHg and/or DBP ≥ 90 mmHg after at least 5 min of supine rest [33]. This condition, properly named neurogenic SH, also represents a marker of cardiovascular dysautonomia and might be particularly severe at night-time when the subject maintains the supine position for several hours (nocturnal hypertension). Twenty-four hour blood pressure monitoring in such patients shows a loss of the expected nocturnal blood pressure fall at night (by 10–20% compared to mean daytime values). Typical blood pressure profiles observed in this context are characterized by a reduced-dipping pattern (less than 10%) or non-dipping or reverse dipping (i.e. an increase with respect to daytime).

It is important to remember that a reduced-dipping pattern or nocturnal hypertension may be observed outside the context of autonomic failure, most notably in patients with essential hypertension. These patients, differently from patients with cardiovascular autonomic failure (NOH and SH) show hypertension both in the supine position and while standing. Therefore, presence of hypertension in the supine position or nocturnal hypertension not associated with NOH is not a marker of cardiovascular dysautonomia and should be interpreted otherwise.

So far, no study specifically addressed the topic of neurogenic SH.

Schmidt et al. reported hypertension in supine position in 25% of PSP patients, based on rest values before HUTT [14]. However, it is not known whether these same patients also had OH. The same authors subsequently examined the 24 h blood pressure profile by means of ambulatory monitoring and found night-time hypertension in 36%, a reduction of the expected blood pressure night fall in 40% and a reverse dipping pattern in 8% of PSP patients [21, 22]. Statistical analysis showed a significant correlation between increased blood pressure night-time values and presence of OH at HUTT (74% of patients with a paradox nocturnal blood pressure increase also presented OH at HUTT, whereas only 11% of patients without OH had a reversed nocturnal blood pressure profile) [21]. Nonetheless, it was not specified whether OH had a neurogenic origin. In other words, it is not possible to rule out other causes for these findings (i.e. essential hypertension and OH secondary to medications) even though patients treated with anti-hypertensive medications were excluded from the study which makes this hypothesis unlikely.

Liu et al. reported hypertension in supine position in 61% of PSP patients (based on rest values before standing) and a reduced dipping nocturnal blood pressure profile in 32% and a reverse dipping profile in 32% of patients [17]. In this study, none of the patients presented OH, while 55% were on medication for hypertension, hence it is likely that such alterations may be related to essential hypertension.

PSP had been associated with essential hypertension particularly in the pre-symptomatic phase. One retrospective study found that a history of hypertension on medical records or reported by patients was present in 80% of the PSP patients, a number significantly higher than what encountered in other parkinsonian disorders [34]. Nevertheless, subsequent works including one with pathological confirmation failed to confirm such results, finding that the prevalence of hypertension in PSP was similar to the general population [35, 36]. However, Sibon et al. pointed out the possibility that cases with “mixed” vascular and neurodegenerative pathologies might be more frequent than expected and might have been missed by studies focusing on “pure” neurodegenerative PSP [37]. This topic was again addressed in a large multicentre study on 277 PSP patients compared to 277 age- and sex-matched controls [38]. The Authors found that hypertension (confirmed by use of anti-hypertensive medication) preceding at least 10 years the onset of PSP symptoms was 1.5 times more common in PSP than controls, hence suggesting an association (and not co-occurrence). The Authors hypothesize that hypertension may potentiate the development of PSP through central mechanisms leading to protein aggregation and neuroinflammation, whereas a cerebrovascular component as suggested in previous works seems unlikely.

OH aside, cardiovascular sympathetic and parasympathetic functions were further investigated by means of autonomic testing in 16 studies (15 cross-sectional and 1 retrospective with pathological confirmation). Sympathetic function was mainly derived from the entity of blood pressure drop on orthostatic test and blood pressure responses to Valsalva manoeuvre and isometric exercise, whereas parasympathetic activity was based on HR variability during the Valsalva manoeuvre, deep breathing or orthostatic test. While an overt autonomic failure was never found, some studies concluded for a certain degree of autonomic impairment involving both branches [8, 14, 20–22, 39–43] in contrast to others that found substantial intact responses [18, 19, 44–46]. The average change in SBP with standing in PSP compared to a control group was variable among studies. As a matter of fact, some studies detected a drop in SBP on standing that was significantly different compared to controls such as in the work by van Dijk et al. (PSP = 15.8 ± 11.7 mmHg; controls = 11.2 ± 10.9 mmHg)[39] while others found comparable results, for example Kimber et al. (PSP = 1 ± 2 mmHg; controls = 3 ± 2 mmHg) [18]. Van Gerpen et al. deduced that adrenergic system is relatively preserved in PSP, whereas indices of cardiovagal function could be abnormal similarly to other parkinsonian syndromes (MSA, DLB) [23].

Neuro-hormonal studies based on plasma levels of cathecolamines provided conflicting results suggesting either a normal, increased or impaired sympathetic activity. Plasma levels of norepinephrine at rest and in response to HUTT resulted within normal limits [46]. Conversely, another study found significantly increased levels of norepinephrine at rest in PSP compared to controls [19], suggesting sympathetic hyperactivity. Lower values of adrenaline in response to clonidine challenge test were observed in PSP compared to controls, but considering that other cardiovascular and neurohormonal responses were substantially normal in PSP this was not considered significant [18].

Most research compared PSP not only to controls but also to other parkinsonian disorders, particularly PD and MSA. While MSA showed a more severe autonomic failure in all studies, the comparison between PSP and PD gave conflicting results. As a matter of fact, indexes of sympathetic function were either equal [13, 19, 20, 40, 46], more [39, 42] or less compromised [14, 22, 45] in PSP compared to PD. Likewise, parasympathetic function in PSP was more impaired [39, 40] or comparable [14, 19, 20, 22, 45, 46] to PD.

Meta-iodo-benzylguanidine (MIBG) myocardial scintigraphy was employed to assess cardiac sympathetic innervation in PSP. Some studies showed considerable normal findings [47–51] whilst another reported a mild decrease of MIBG uptake in a small cohort of 6 PSP subjects [52].

In summary, cardiovascular autonomic function was assessed by few studies with considerable differences regarding methods and results. OH was present in a small proportion of patients or not at all, and cases of OH secondary to medications (like dopaminergic drugs) might have been included. Symptomatic OH represented only a minority and generally there was no correspondence between reported symptoms and objective presence of OH. Thus, it is important to always measure blood pressure to check for OH independently from symptoms which may be misleading. NOH does not seem to be a feature of PSP although only 1 study specifically addressed it, with autopsy proven diagnoses which strengthened it.

Supine and nocturnal hypertension were not uncommon in PSP, however it is likely that these findings are mainly related to essential hypertension rather than neurogenic.

Sympathetic and parasympathetic function resulted impaired to a certain degree or substantially normal, whereas a severe impairment was never described.

While PSP autonomic function is generally more preserved compared to MSA patients, the differences with PD were less clearly defined. Distinguishing PSP from PD at an early stage, when the full clinical picture has not developed yet, may be challenging and finding elements that may help identify one condition rather than another is of the utmost importance, especially as new disease-modifying treatments are being developed. Based on the studies performed so far, cardiovascular autonomic function does not seem to be useful for this purpose. However, such studies were either cross-sectional with disease duration between 3 and 5 years and without data from very early stages. Therefore, prospective studies from disease onset with regular and long follow-up that would better provide answers to this crucial topic are needed. It is likely that rather than a single test, the complementary information derived from the combination of different tests performed at the same time might serve better to this purpose.

### Urogenital system

Lower urinary tract symptoms (LUTS) are usually classified into irritative (nocturia, frequency, urgency with or without incontinence) and obstructive (delay in initiating micturition, slow stream, straining to void, incomplete bladder emptying, retention). The former mainly reflect an impairment of the storage function of the bladder, whereas the latter a hindrance in the voiding phase. Even though urinary complaints are very common in PSP, the number of studies assessing them is relatively scanty. Overall the prevalence of LUTS is considerably high, reaching 93% [7, 9, 10, 14, 15, 22]. In the majority of studies irritative symptoms were reported more frequently (46–89%) than obstructive ones (40–56%) [8, 53–55], with nocturia being the commonest [7, 13]. However, Reimann and colleagues found obstructive symptoms (problems to initiate micturition) in a higher number of patients compared to irritative complaints like urgency and incontinence (prevalence 63% vs 37% and 48% respectively)[22]. In addition, nocturia was uncommon in PSP in Kim and colleagues’ work, with only 10% of the patients reporting it [55]. Some studies addressed only irritative symptoms, finding similar prevalence figures (53–81%) [11, 16, 24]. Importantly, early development of urinary incontinence was associated with poor prognosis [24, 56]. Subjective urinary dysfunction correlated with disease severity (assessed by the PSP rating scale, PSPRS, and unified PD rating scale, UPDRS) [7, 57] and affected quality of life [57].

These studies are very heterogeneous regarding the design (cross sectional vs retrospective without or with pathological confirmation) and methods of LUTS assessments (review of medical records, clinical interview, different types of questionnaires and scales not specifically validated for PSP). Such tools may have the advantages of being easily applicable even to a large number of subjects and exploring a wide range of symptoms, nevertheless they do not take into account possible other causes of urinary symptoms (i.e. they are not specific enough for urinary symptoms due to autonomic dysfunction). As a matter of fact, other conditions related to age may influence urinary function, for instance prostate hypertrophy and stress incontinence from weakness of pelvic floor muscles to name a few.

Most studies compared scores from PSP with those of other parkinsonisms, namely PD and MSA. Compared to PD, total urinary scores of PSP were equal [9, 12, 13, 57] or significantly higher [7]. Irritative symptoms were found to be similar [9, 54, 55], higher [7, 22] or lower (only nicturia) [55] in PSP compared to PD, while obstructive either similar[13, 54, 55] or higher[22]. Likewise, compared to MSA, total urinary score of PSP were equal [12, 57] or significantly lower [13]. Irritative symptoms were similar [54, 55] or lower [22], whilst obstructive higher [22], similar [55] or lower [13, 54]. The same limitations mentioned above limit the possibility of comparing these contrasting results among studies.

Urodynamic studies allow the objective quantification of bladder function regarding both the storage and voiding phase, and can therefore identify bladder dysfunction due to neurogenic origin. Urodynamic studies are performed in specialized laboratories and require a quite invasive technique by inserting a first catheter in the bladder to monitor intravesical pressure and a second catheter inside the rectum or vagina to record intra-abdominal pressure. Very often, EMG of the external urethral or rectal sphincter is also performed. Pressures, volumes and detrusor contractions during filling and voiding are then recorded, along with corresponding symptoms reported by the patient (awareness of bladder distension, urge to urinate, etc.).

Only 3 studies performed full urodynamic evaluations in PSP (1 retrospective, 2 cross-sectional) and all found both storage and voiding abnormalities. With regard to storage phase, detrusor hyperreflexia was detected in 67 to 90% of the patients [53–55], along with reduced volumes at first desire or urge to void and reduced bladder compliance in 17–67% [53]. Detrusor-external sphincter dyssynergia, which marks an abnormal voiding phase, was observed in 10–17% of PSP individuals [53–55]. An increased post-void residual urine volume (> 100 mL) was found in only 1 out of 6 patients [53]. This very low prevalence was confirmed by the work of Lee and colleagues who explored post-void residual volume with an ultrasound device (1 out 19 PSP patients had abnormal post-void residual volume defined as > 100 mL) [57]. Therefore, urodynamic studies seem to corroborate the higher representation of storage phase dysfunction compared to voiding phase as suggested by questionnaire general results.

A report of a single patient with a clinical diagnosis of PSP described marked urinary retention with the need for permanent catheterization within 3 years from motor onset [58]. Urodynamic assessment showed storage abnormalities but predominant voiding dysfunction with severe detrusor hypoactivity. However, the single nature of this case and the lack of pathological confirmation limit the accuracy of these data.

Urodynamic measures do not seem to correlate with other clinical parameters, except that disease duration of PSP with detrusor overactivity was longer than PSP without detrusor overactivity [54] and between older age and involuntary detrusor contraction during filling cystometry [55].

Urodynamic parameters were compared between PSP and other parkinsonian syndromes. Yamamoto et al. [54] found that PSP had higher detrusor overactivity than PD and MSA whereas detrusor-sphincter dyssynergia in PSP was higher than PD but lower than MSA, however without reaching statistical significance in neither case. In the same study, post-void residual urine volume in PSP (105 ± 18 mL) was significant larger compared to PD (40 ± 3.8 mL) and equivalent to MSA (113 ± 7.5 mL). The lack of significant difference between PSP and MSA may be surprising considering that, as mentioned earlier, an abnormal post-void residual volume was uncommon in PSP whereas urinary retention is a major feature of MSA, being also listed among the diagnostic criteria [59]. This finding may be explained by the fact that the mean post-void residual volume in MSA patients considered in this study was relatively low compared to mean values usually observed in this disease. Indeed, Kim and colleagues found a larger post-void residual volume in MSA (233 ± 192 mL) compared to PSP (180 ± 219 mL), which was in turn larger compared to PD (90 ± 123 mL) although without reaching statistical significance. Conversely, the Authors did not find any significant difference in storage phase parameters [55].

Studies assessing urinary autonomic function are listed in Table 2.

**Table 2:** Studies assessing urinary function and symptoms in PSP

References	Study design	N° PSP (m/f)	Age (y)	Disease duration (y)	Assessment	Symptoms	Storage abnormalities	Voiding abnormalities	PMV
Wenning et al. [16]	Retrospective + Pathology	24 (15/9)	63.1 ± 6.9 (onset)	79 ± 44 (months)	Review of medical records	Incontinence (75%)			
Sakakibara et al. [53]	Retrospective	9 (6/3)	67.3 ± 4.7	4.6 ± 2.5	Detailed clinical history, urodynamic study	Irritative (89%), obstructive (44%)	Detrusor hyperreflexia (67%), reduced volume at first desire to void (17%), reduced volume at urge to void (67%), reduced compliance (17%)	DSD (17%)	Increased (17%)
Donker-Kaat et al. [56]	Prospective	152	66.8 7.9 (onset)	5.4 ± 2.7	Review of medical records	Incontinence			
Schmidt et al. [14]	Cross-sectional	32 (22/10)	66 ± 7	3.5	Standardized clinical interview	All urinary symptoms (93%)			
Bae et al. [13]	Cross-sectional	21 (11/10)	69.4 ± 6.9	3.5 ± 2.1	Autonomic dysfunction questionnaire	Both irritative and obstructive			
Reimann et al. [22]	Cross-sectional	32 (22/10)	65.8 ± 6.9	3.5	Autonomic dysfunction score (interview)	Irritative (37–48%), obstructive (63%)			
Colosimo et al. [11]	Longitudinal	30 (16/14)	69.7 ± 6.3	3.5 ± 3.1	Interview	Irritative (53%)			
Tateno et al. [58]	Case report	1 (f)	62	3	Clinical history	Retention	Yes	Yes	Increased
Berganzo et al. [12]	Cross-sectional	20 (11/9)	68.3 ± 7.2	5.0 ± 2.4	SCOPA-Aut	Mean total score 37.72			
Yamamoto et al. [54]	Retrospective	47 (39/8)	71.4 ± 0.88	2.97 ± 0.26	Authors’ original questionnaire, urodynamic study	Irritative (57%), obstructive (56%)	Detrusor hyperreflexia (81%)	DSD (13.9%)	Increased
Lee et al. [57]	Cross-sectional	19 (9/10)	75.6 ± 7.6	0.6 ± 0.9	SCOPA-Aut, ultrasound measurement of PMV				Increased (5%)
Ou et al. [9]	Case–control	27 (16/11)	65.1 ± 8.4	3.6 ± 2.1	NMSS	All symptoms (74.1%)			
Radicati et al. [7]	Cross-sectional	50 (22/28)	69.8 ± 9.0	3.8 ± 2.1	NMSS	Nocturia 82%, frequency 68%			
Kim et al. [55]	Cross- sectional	10 (9/1)	71.9 ± 6.1	3.0 ± 1.7	Clinical interview, urodynamic study	Irritative (10–70%), obstructive (10–30%)	Detrusor hyperreflexia (90%)	DSD (10%)	Increased
Oliveira et al. [24]	Retrospective + Pathology	103 (64/39)	66.8 [62.1–72.5] (onset), 74.6 [70.4–81.4] (death)	n/a	Review of medical records	Irritative (81%)			
Nojszewska et al. [8]	Cross-sectional	37	67.5 ± 6.1	4.6 ± 3.6	Modified Autonomic Symptom Questionnaire	Incontinence (46%), retention (40%)			
Chaithra et al. [10]	Cross-sectional	76 (53/23)	62.04 ± 7.10	2.68 ± 2.10	NMSS	Irritative (53.9%)			
Dubbioso et al. [15]	Cross-sectional	27 (18/9)	70 ± 7.4	3.6 ± 2.1	SCOPA-Aut SFN-SIQ	81% scored ≥ 2 in at least 1 item of urinary domain Micturition disturbances (85%)			

*DSD* detrusor-sphincter dyssynergia, *NMSS* Non-Motor Symptom Scale, *PMV* post-micturition volume, *SCOPA-Aut* Scales for Outcomes in Parkinson’s Disease-Autonomic, *SFN-SIQ* small fiber neuropathy symptoms inventory questionnaire

Sexual function has been investigated in very few studies each employing a different method. Erectile dysfunction was reported in the medical records of 31% of autopsy-proven PSP [24]. Other studies using distinct questionnaires found impotence in around 50% [8, 15] and change of libido as most frequently reported sexual symptom [13]. Prevalence of sexual dysfunction symptoms investigated by means of the NMSS was 18% [7], 70% [10] and 93% [9].

Erectile dysfunction had a higher frequency in PSP compared to healthy control subjects [24]. However, sexual dysfunction resulted comparable between PSP and both PD and MSA [7, 8, 12, 13, 24] but for one study that found significantly higher prevalence in PSP compared to PD[9]. One study found a higher total score for the sexual domain in PSP compared to DLB [13].

Finally, earlier development erectile dysfunction did not influence survival [24].

In summary, urinary complaints are very common in PSP, particularly irritative symptoms/storage phase dysfunction, which were confirmed by urodynamic studies showing high prevalence of detrusor hyperreflexia. Importantly, early development of urinary incontinence impacts negatively prognosis. Urinary symptoms do not seem to be useful to differentiate PSP from PD or MSA. An increased post-void residual urine volume may distinguish PSP from PD but not from MSA, however this is based on the observations of very few studies conducted on a small number of patients. The paucity of studies addressing this topic and the lack of prospective observation make our current knowledge on urinary autonomic dysfunction limited.

### Gastrointestinal system

Gastrointestinal problems are notoriously well recognized in parkinsonisms, and PSP is no exception. Symptoms and signs regarding this domain are various, starting from dysphagia to oesophageal dysmotility, stomach (gastroparesis, bloating, nausea and vomiting) and intestinal problems (constipation, diarrhoea). One more time, there is a paucity of studies focusing on this topic, and the few available use heterogeneous methods.

In general, 80–89% of PSP patients reported symptoms of gastrointestinal dysfunction [7, 11, 14, 22] even though some studies found lower prevalence (66% [10], 52% [15] and 40% [9]). The most common features were constipation [14], dysphagia [7, 10, 13] or both [22].

In the retrospective work with pathological confirmation by Oliveira and colleagues, constipation was recorded in 71% of PSP patients, and an earlier development was associated with shorter survival [24]. Similarly, a recent meta-analysis found that early dysphagia was an unfavourable predictor of survival in patients with PSP-Richardson syndrome phenotype [60].

Gastrointestinal score on the NMSS significantly correlated with results from the PSP rating scale (total and single domain scores) in one study [7].

Compared to PD, gastrointestinal scores of PSP did not differ significantly [12, 14] or were higher [7, 9, 13, 22]. Instead, Oliveira et al. found a significantly higher prevalence of constipation in PD than PSP [24]. Similarly, compared to MSA, gastrointestinal symptoms in PSP were equal [8, 12, 22] or more frequent [13].

Studies applying instrumental objective techniques to study the gastrointestinal system mainly targeted swallowing function. Videofluorographic swallowing assessment in 51 PSP patients (the study with the largest numerosity)[61] showed that the oral phase was more severely impaired than the pharyngeal phase, confirming previous findings [62–65]. This suggests that swallowing difficulties in PSP could be mainly attributed to other circuits than autonomic. Indeed, neuroimaging correlates of oral dysphagia in PSP were localized in cortical motor control centres [66].

Claus and co-workers investigated oesophageal function by means of high-resolution manometry in 10 patients with PSP [67]. The Authors found that peristalsis measures and magnitude of distal oesophageal contraction in PSP were within normal ranges and higher compared to MSA and PD, who instead showed a trend towards oesophageal hypomotility. This seems to support preserved oesophageal function in PSP, which depend upon peripheral autonomic mechanisms. However former studies detected reduced oesophageal peristalsis in 2 out of 25 PSP patients [62], diffuse oesophageal spasm as well as hypertensive or inadequate relaxation of the upper/lower oesophageal sphincters in 6 out of 7 PSP [63] and multiple abnormalities of oesophageal motility in all the 10 PSP patients examined [68]. These studies however involved patients with longer disease duration than those in the study by Claus et al. and were performed several years previously, therefore it is possible that the procedures had different resolutions.

Bowel sounds, reflecting intestinal motility, were recorded by a digital auscultation system that provided a quantitative analysis in 5 PSP, 10 PD and 12 MSA [69]. Bowel sounds in PSP did not differ significantly to those of controls, instead PD and MSA patients demonstrated reduced bowel sounds which reached statistical significant difference only compared to controls (not PSP).

Colonic transit time was assessed by the repetitive ingestion method in 8 PSP, 36 PD and 8 controls [70]. While not reaching statistical significance, PSP and PD presented a trend towards prolonged total colonic transit time. The right colonic transit time was significantly longer in PSP and PD compared to controls. This finding may suggest a possible involvement of the vagal nerve that innervates the right colon.

Surgical sigmoid colon specimens of 5 PSP, analysed by Western Blot and immunochemistry using a panel of anti-tau antibodies, showed neither abnormal phosphorylation nor truncation of tau [71]. It may be speculated that the tau-related pathological process affecting the CNS in PSP does not involve also the enteric nervous system, as it happens with synucleinopathies (particularly PD).

Studies assessing gastrointestinal function are reported in Table 3.

**Table 3:** Studies assessing other autonomic domains in PSP

References	Study design	N° PSP (m/f)	Age (y)	Disease duration (y)	Assessment	Results
Gastrointestinal						
Litvan et al. [62]	Cross- sectional	27 (18/9)	64.9 ± 1.2	52 ± 5 (months)	Swallowing questionnaire, videotaped ultrasound imaging study of swallowing, videotaped modified barium swallow study	Significant abnormalities of swallowing, 2 patients had reduced esophageal peristalsis
Johnston et al. [63]	Case–control	7 (5/2)	70	5	Barium swallow; manometry of esophagus and pharynx	Barium: pooling in either pyriform sinuses or valleculae commonest abnormality Manometry: diffuse esophageal spasm most frequent abnormality, inadeguate relaxation of the UES during swallowing
Leopold and Kagel [68]	Cross-sectional	10 (7/3)	70.9 ± 2.23	4.1 ± 2.38	Dynamic videofluoroscopic swallowing function study	Multiple abnormalities of esophageal motility: tertiary contractions, sphincters abnormalities
Alfonsi et al. [64]	Cross-sectional	9 (4/5)	71 [64–77]	4 [3–6]	EMG study of swallowing	Dysphagia was early and not correlated with UPDRS. EMG: prolonged LPM-D; absence of EMG silence of the cricopharyngeal muscle
Schmidt et al. [14]	Cross-sectional	32 (22/10)	66 ± 7	3.5	Standardized clinical interview	All symptoms (89%), constipation most frequent
Bae et al. [13]	Cross-sectional	21 (11/10)	69.4 ± 6.9	3.5 ± 2.1	Autonomic dysfunction questionnaire	Dysphagia most frequent symptom
Reimann et al. [22]	Cross-sectional	32 (22/10)	65.8 ± 6.9	3.5	Autonomic dysfunction score (interview)	All symptoms (89%), constipation and dysphagia (52%)
Warnecke et al. [65]	Cross-sectional	18 (11/7)	69.67 ± 8.99	3.47 ± 1.89	FEES	Abnormal findings in all main endoscopic parameters of swallowing function
Colosimo et al. [11]	Longitudinal	30 (16/14)	69.7 ± 6.3	3.5 ± 3.1	Interview	All symptoms (80%)
Ozawa et al. [69]	Cross-sectional	5	73.1 ± 5.7	2.6 ± 1.6	Digital auscultation system of bowel sounds	No differences with controls
Berganzo et al. [12]	Cross-sectional	20 (11/9)	68.3 ± 7.2	5.0 ± 2.4	SCOPA-Aut	Mean total score 22.81
Ou et al. [9]	Case–control	27 (16/11)	65.1 ± 8.4	3.6 ± 2.1	NMSS	Total symptoms (40.4%)
Radicati et al. [7]	Cross-sectional	50 (22/28)	69.8 ± 9.0	3.8 ± 2.1	NMSS	All symptoms (86%), swallowing (64%), constipation (58%)
Claus et al. [67]	Cross-sectional	10 (9/1)	72.6 ± 3.8	2.5 ± 0.9	High-resolution manometry and FEES	Esophageal peristalsis preserved
Oliveira et al. [24]	Retrospective + Pathology	103 (64/39)	66.8 [62.1–72.5] (onset), 74.6 [70.4–81.4] (death)	n/a	Review of medical records	Constipation (71%), earlier development was negative prognostic factor
Nojszewska et al. [8]	Cross-sectional	37	67.5 ± 6.1	4.6 ± 3.6	Modified Autonomic Symptom Questionnaire	Constipation (41%), dysphagia 32%)
Clark et al. [61]	Cross-sectional	51 (26/21)	71 [65–75]	4 [2–5]	Standardized clinical interview, videoflurographic swallowing assessment	Two thirds of patients had swallowing abnormalities
Doi et al. [70]	Cross-sectional	8 (6/2)	74.6 ± 8.5	2.9	Pelvic organ questionnaire, CCT test (Devroede’s repetitive ingestion method)	25% reported constipation. Trend for a prolonged total CTT in PSP that however did not reach statistical significance while right CCT was significantly prolonged
Chaithra et al. [10]	Cross-sectional	76 (53/23)	62.04 ± 7.10	2.68 ± 2.10	NMSS	Total symptoms (65.8%)
Dubbioso et al. [15]	Cross-sectional	27 (18/9)	70 ± 7.4	3.6 ± 2.1	SCOPA-Aut SFN-SIQ	52% scored ≥ 2 in at least 1 item of gastrointestinal domain Gastroparesis 11%, constipation/diarrhoea 56%
Sudomotor						
Sandroni et al. [40]	Retrospective	32 (19/13)	65.6 ± 6.6 (at onset)	n/a	TST, QSART	TST: anterior anhidrosis (50%) QSART: abnormalities forearm (50%), abnormalities foot (58%)
Kikkawa et al. [19]	Cross-sectional	12 (9/3)	70.4 ± 6.3	3.7 ± 2.8	SSR (sympathetic activation test e.g. mental arithmetic)	SSR absent (58%)
Schmidt et al. [14]	Cross-sectional	32 (22/10)	66 ± 7	3.5	Standardized clinical interview	All symptoms (59%), nocturnal sweating most frequent
Bae et al. [13]	Cross-sectional	21 (11/10)	69.4 ± 6.9	3.5 ± 2.1	Autonomic dysfunction questionnaire	Heat intolerance most frequent symptom
Reimann et al. [22]	Cross-sectional	32 (22/10)	65.8 ± 6.9	3.5	Autonomic dysfunction score (interview), SSR (acustic stimulus)	All symptoms (59%); SSR absent (48%)
Colosimo et al. [11]	Longitudinal	30 (16/14)	69.7 ± 6.3	3.5 ± 3.1	Interview	Hyperhidrosis and seborrhoea (17%)
Berganzo et al. [12]	Cross-sectional	20 (11/9)	68.3 ± 7.2	5.0 ± 2.4	SCOPA-Aut	Mean total score 10.09
Nojszewska et al. [8]	Cross-sectional	37	67.5 ± 6.1	4.6 ± 3.6	Modified Autonomic Symptom Questionnaire, SSR (electrical stimnulus)	SSR absent or longer latencies (70%)
Van Gerpen et al. [23]	Retrospective + Pathology	14 (7/7)	68 [40–77] (onset), 74 [54–91] (death)	8 [4–15]	CASS, QSART	50% CASS sudomotor score 2
Dubbioso et al. [15]	Cross-sectional	27 (18/9)	70 ± 7.4	3.6 ± 2.1	SCOPA-Aut SFN-SIQ SSR (electrical stimulus) Dynamic sweat test	44% scored ≥ 2 in at least one item thermoregulatory domain 30% hyperhidrosis/hypohydrosis Reduced SSR amplitude Reduced sweat output
Pupillomotor						
Nath et al. [73]	Prospective	187 (89/98)			Review of medical records for photophobia	43%
Schmidt et al. [75]	Cross-sectional	24 (18/6)	64 ± 7	3.5 (2.8; 6.3)	Pupillography	71% pathological pupil diameter in darkness in at least 1 eye, pupil light reflex preserved
Cooper and Josephs [74]	Prospective + pathology (2)	10 (4/6)	66 [59–77] (onset)	n/a	Clinical history of photophobia	100%
Reimann et al. [22]	Cross-sectional	32 (22/10)	65.8 ± 6.9	3.5	Autonomic dysfunction score (interview), pupillography	All symptoms (85%), 68% pathological pupil diameter in darkness
Berganzo et al. [12]	Cross-sectional	20 (11/9)	68.3 ± 7.2	5.0 ± 2.4	SCOPA-Aut	Mean total score 28.07
Dubbioso et al. [15]	Cross-sectional	27 (18/9)	70 ± 7.4	3.6 ± 2.1	SCOPA-Aut SFN-SIQ	41% scored ≥ 2 in at least one item of pupillomotor domain 48% reported accommodation disturbances
Secretomotor and vasomotor						
Kikkawa et al. [19]	Cross-sectional	12 (9/3)	70.4 ± 6.3	3.7 ± 2.8	Skin vasomotor reflex	Normal
Schmidt et al. [14]	Cross-sectional	32 (22/10)	66 ± 7	3.5	Standardized clinical interview	All vasomotor symptoms 59%, cold hands most frequent All secretomotor symptoms 78%, hypersalivation most frequent
Reimann et al. [22]	Cross-sectional	32 (22/10)	65.8 ± 6.9	3.5	Autonomic dysfunction score (interview)	All vasomotor symptoms 59%, cold hands most frequent All secretomotor symptoms 81%, hypersalivation most frequent
Schubert et al. [51]	Cross-sectional	25 (13/12)	69 ± 7	5.6 ± 2.7	MIBG scintigraphy salivary glands	Normal uptake
Dubbioso et al. [15]	Cross-sectional	27 (18/9)	70 ± 7.4	3.6 ± 2.1	SFN-SIQ	41% reported sicca syndrome

*CASS* Composite Autonomic Severity Score, *CCT* colonic transit time, *FEES* fiberoptic endoscopic evaluation of swallowing, *LPM-D* duration of laryngeal-pharyngeal mechanogram, *MIBG* meta-iodo-benzylguanidine, *NMSS* Non-Motor Symptom Scale, *QSAR* quantitative sudomotor axon reflex test, *SCOPA-Aut* Scales for Outcomes in Parkinson’s Disease-Autonomic, *SFN-SIQ* small fiber neuropathy symptoms inventory questionnaire, *SSR* sympathetic skin response, *TST* thermoregulatory sweat test, *UPDRS* Unified Parkinson’s disease rating scale

In summary, symptoms of gastrointestinal dysfunction are reported quite commonly in PSP, particularly dysphagia and constipation. Dysphagia seems to mainly derive from an impairment of the oral phase, which is a complex motor event depending on cortical and subcortical circuit activation with limited involvement of the autonomic nervous system. However, progressive degeneration of brainstem nuclei that are part of the central pattern generators of swallowing and control the pharyngeal/reflex phase may play a role. Instead, esophageal motility, more strictly dependent on autonomic function, seems to remain preserved in early stages. Data on intestinal peristalsis were inconclusive.

Considering the high frequency of reported symptoms, there are very few studies objectively assessing the function of the digestive tract in PSP, with the few available so far performed on an exiguous number of patients.

### Sudomotor function

Sweat glands, located in the skin throughout the body although with regional differences, are richly supplied by sympathetic nerve fibres which are characteristically cholinergic. The main function of the sweat glands is thermoregulatory.

Symptoms regarding the sudomotor domain were reported by 59% of PSP patients [14], with nocturnal sweating [22] and heat intolerance [13] being the commonest. Another study using a structured interview found a prevalence of 17% of skin problems that included hyperhidrosis and seborrhoea [11].

Sweating disturbances reported by questionnaires did not seem to differ between PSP and other parkinsonisms [12–14, 22]. However, one recent study suggested that PSP showed thermoregulatory dysfunction at the SCOPA-Aut more frequently than PD (44% vs 24%) [15].

Sudomotor function can be assessed objectively by means of several tests that specifically investigate different aspects. The Quantitative sudomotor axon reflex test (QSART) evaluates the activity of postganglionic fibres. The stimulus elicited in the postganglionic sympathetic terminal travels antidromically to reach a branch-point and then goes orthodromically to release acetylcholine from a nerve terminal. The sweat response to this test consists in the sweat volume which is measured by a sudorometer. Because the response may be physiologically influenced by age and gender, Low and colleagues developed the CASS to correct for such confounding factors. The CASS sudomotor score ranges from 0 (no deficit) to 3 (maximal deficit). The thermoregulatory sweat test (TST) assesses both the preganglionic (including also the pre-autonomic neurons in the hypothalamus) and postganglionic paths. The subject is unclothed and covered with a powder that function as an indicator for sweating, then placed in a closed cabinet with rising temperature. The sweat distribution is documented by digital photography and the percentage of anhidrosis calculated. The sympathetic skin response (SSR) evaluates skin potentials that are known to correlate to sudomotor function. The SSR is performed by providing a stimulus (which may be of varying nature, e.g. acoustic, electric, mental stress) and recording skin potentials via two electrodes usually applied on the ventral surface of the foot or hand. The absence of SSR is abnormal whereas a reduction in the amplitude or latency of the potential is more difficult to interpret. This test is relatively simpler to perform than the formers however it is subjected to greater variability depending on the equipment, type of stimulus and interpretation, which limits its standardization and possibility of comparison across studies.

A handful of studies employed these techniques to study sudomotor function in PSP.

Sandroni et al. found pathological QSART responses in approximately 50% of PSP [40]. Similarly, a CASS sudomotor score of 2 was found in 50% of patients with a pathologically-proven PSP diagnosis [23]. Recently, autonomic sudomotor function was assessed in 27 PSP patients with the dynamic sweat test, which evaluates sweating output using the same principles of the QSART. All patients presented a marked reduction of activated sweat gland density and sweat output/cm^2^ [15].

PSP showed 50% of anterior anhidrosis at the TST [40].

The SSR was pathological (absent) in 48% [22] and 58% [19] of PSP. Another study reported impaired SSR (absent or longer latencies) in 70% of patients [8]. SSR amplitude was significantly lower in PSP compared to healthy controls [15].

Regarding the comparisons with other extrapyramidal syndromes, there was no significant difference in the CASS sudomotor subscore between PSP, MSA and LBD, suggesting that dysautonomia involving the sudomotor domain is represented across all parkinsonisms [23]. The percentage of anhidrosis at the TST in PSP was intermediate between PD (lower) and MSA (higher) [40].

The SSR in PSP was found to be similar to MSA and PD[22] and more impaired than MSA [8] and PD [15, 19].

The list of studies on sudomotor function can be found in Table 3.

In summary, sudomotor dysfunction is not uncommon in PSP. Roughly half of PSP patients report symptoms of sudomotor dysfunction, which seems to be confirmed by objective measures that actually in some cases disclosed abnormalities even in higher percentages of patients, possibly reflecting a lack of awareness for these disturbances. However only very few studies are available on this interesting topic.

### Pupillomotor function

The autonomic nervous system regulates pupil size at rest and in response to several stimuli. The parasympathetic branch induces miosis as a reaction to light and accommodation whilst the sympathetic promotes mydriasis in response to its activating factors. A dysfunction of the autonomic nervous system innervating this area causes altered pupil size at rest and/or in response to stimuli and possibly abnormal shape. The pupillography technique allows an objective determination of the pupil size and characteristics of pupillary responses, particularly the light response which is the easiest and safest to perform.

Symptoms of pupillomotor dysfunction are quite common in PSP. Indeed, photophobia is listed among the supportive features in the latest diagnostic criteria [3]. Photophobia and other visual symptoms like blurred vision and difficulty in focusing are known to occur even in early stages of PSP [72, 73]. One study found an 85% prevalence of reported pupillomotor symptoms, with light hypersensitivity being the most frequent (67%) [22]. In another study photophobia was present in 100% of PSP cases, however it was based on a small group of 10 patients [74]. The same study suggested that given the low occurrence of this symptom in corticobasal syndrome (CBS) (18%), photophobia may be a useful feature to differentiate these two entities in clinical practice. In a more recent study, pupillomotor dysfunction was described in a lower percentage of PSP patients (41–48%)[15]. Interestingly, some patients included in this study had a PSP-CBS phenotype. It is possible that pupillomotor symptoms are a characteristic feature of the Richardson syndrome phenotype and hence including other phenotypes may reduce their prevalence. Visual symptoms were not significantly different between PSP and PD/MSA [12, 22], however one study found a higher percentage of pupillomotor dysfunction in PSP compared to PD (41% vs 9%) [15].

Only two studies, carried out on the same cohort of PSP patients, performed pupillography to objectively evaluate pupillary function. The diameter of pupils after darkness adaptation was pathological in at least one eye in approximately 70% of PSP patients and this figure was significantly higher than PD and MSA [22, 75]. On the contrary, no significant differences were found in the light reflex between these parkinsonian groups [75].

Studies on pupillomotor function are listed in Table 3.

In summary, visual symptoms suggesting pupillomotor dysfunction are common and a characteristic feature of PSP, particularly the Richardson syndrome phenotype. Surprisingly however only one group performed objective assessment of pupil function, suggesting a potential discriminating role between PSP and other parkinsonisms. Further studies should be encouraged to confirm such findings.

### Secretomotor and vasomotor function

These aspects of autonomic function have been poorly assessed in PSP. Only two studies performed by the same group investigated by means of a structured questionnaire these symptoms. Vasomotor symptoms were reported by 59%, the most frequent item was cold hands which was present in 44% of patients [14, 22]. Approximately 80% of the patients reported secretomotor symptoms, particularly hypersalivation (63%) and increased lacrimation (44%) in contrast to dry mouth which was relatively infrequent (22%) [14, 22]. Conversely, dry mouth/eyes were found in 41% of PSP patients in another study [15].

These features were comparable between PSP, PD and MSA, with the exception of increased lacrimation which was significantly more frequent in PSP with respect to MSA and dry mouth which was viceversa more frequent in MSA with respect to PSP [22].

Skin vasomotor reflex was objectively studied in the work by Kikkawa and colleagues who recorded cutaneous blood flow on the right index finger by Doppler flowmeter following sympathetic activation stimuli (mental arithmetic, exercise, deep inspiration, tactile sensation) and calculated the reduction rate of blood flow below basal value. The results were within normal values and no significantly different from healthy controls, suggesting that cutaneous vasomotor function may be relatively spared in PSP [19].

One study evaluated MIBG scintigraphy of the salivary glands in PSP patients, finding normal results in 22 out of 25 individuals, suggesting preservation of sympathetic innervation in these organs [51].

Studies on secretomotor and vasomotor function are reported in Table 3.

In summary there is a paucity of studies addressing vasomotor and secretomotor functions in PSP. Symptoms referred to this domain are reported in more than half of the patients, particularly increased lacrimation and salivation. Cutaneous vasomotor function seems to be preserved.

## Conclusions

In this review we provide an overview of the current literature investigating the autonomic nervous system involvement in PSP. While symptoms were reported quite frequently for all autonomic domains in PSP population, objective assessment of autonomic functions did not always provide the same conclusions.

Cardiovascular autonomic testing showed that OH is not frequent in PSP however there may be a certain degree of sympathetic and parasympathetic impairment (never severe). There is some evidence suggesting bladder dysfunction particularly in the storage phase. Dysphagia is very common in PSP but it may be caused by multisystem involvement. Abnormalities in the sudomotor function were found in almost half the patients. Similarly, pupillomotor dysfunction seems quite common in those with Richardson’s phenotype.

There are some considerations regarding these findings. To begin with, it should be discussed whether these symptoms and signs are specific of an involvement of the autonomic nervous system in the context of the PSP tauopathy. Indeed elderly people not affected by a neurodegenerative condition frequently report visceral symptoms. Similarly, OH prevalence in the normal aging population (> 65 years) has been reported around 5 and 30% [76]. Age-related changes to the baroreflex due to atherosclerosis, deconditioning due to reduced mobility, drugs and cognitive impairment are all common factors in the elderly that can favour the development of OH. In this regard, performing additional tests such as the Valsalva manoeuvre or at least consider the HR response associated to the orthostatic blood pressure drop may provide more specific information regarding the presence of autonomic nervous system failure.

Further insights can be drawn from few neuropathological studies, which suggest that actually there may be an involvement of the autonomic nervous system related to PSP pathology. One neuropathological study on 17 PSP demonstrated consistent abnormalities in several autonomic nuclei located in the brainstem, namely the medial and parabrachial nuclei, the gigantocellular reticular nucleus, the raphes magnus and raphes obscurus nuclei and intermediate reticular zone, all of which play a role in the regulation of cardiovascular function and/or micturition. Such abnormalities were observed even in cases with short disease duration, however the correlation with autonomic symptoms in life in these patients is unknown [77]. The intermediolateral column in the spinal cord was found to be spared [78] or moderately affected (39% nerve cell loss) by the neurodegenerative process [79], yet none of the patients in the latter study presented signs of autonomic failure in life. Significant pathology was not found in spinal ganglia but sympathetic ganglia were not specifically assessed [80].

Interestingly, peripheral autonomic involvement was observed in skin biopsies from PSP patients that showed a marked reduction in vasomotor, sudomotor and pilomotor nerves [15]. On the other hand, scintigraphic studies showed preserved sympathetic innervation in the myocardium and salivary glands. Mechanisms leading to neurodegeneration of peripheral fibres and the possible selectivity of this process are still to be unravelled.

Another interesting field of study would be to better characterize the premotor phase of the disease. As in PD, non-motor symptoms (including autonomic) may predate the onset of classical feature even by years. Indeed Painous and colleagues in their retrospective study found that PSP patients reported a wide variety of symptoms several years before the diagnosis, including symptoms related to the autonomic nervous system in some cases [81]. However these did not differentiate PSP from PD. Further studies should be encouraged in this regard particularly considering the importance of a precocious diagnosis in light of emerging disease-modifying treatments.

Studying the autonomic nervous system in PSP may have implications also for other domains affected in this disease. For instance, Liu et al. suggested that increased blood pressure variability (that is excessive fluctuations in blood pressure values throughout the day) may influence executive functions in PSP patients, possibly by modifying cerebral perfusion [17]. These preliminary findings are interesting although need to be replicated and supported by neuroimaging findings to better evaluate the causal relation.

There are several limitations regarding the current literature on this topic. Firstly, the majority of studies were based on patient-reported questionnaires and/or scales, implying the assumption that visceral symptoms automatically reflect autonomic nervous system dysfunction. This is incorrect, as the way visceral symptoms are perceived by the individual is the result of complex information processing in the central nervous system [82]. Relying on data from questionnaires and scales may therefore be misleading and not truly indicate autonomic dysfunction.

Secondly, most studies were single-center and performed on a small sample of patients. This may hinder several possible errors related to methods and statistics.

Thirdly, clinical studies mainly recruited patients with PSP-Richardson’s syndrome and as a consequence data on other clinically defined phenotypes are virtually absent. Clinical diagnosis of PSP phenotypes other than Richardson’s syndrome is however challenging and their degree of sensitivity and specificity is lower compared to Richardson’s.

Moreover, prospective studies and those with neuropathological confirmation represent the minority. Therefore, most of the data derive from cross-sectional studies with clinical diagnosis. In general, there is a need for prospective studies who might track different autonomic domains since the earlier stage of the disease.

Lastly, severe autonomic failure is an exclusion criterion for PSP and hence the studies that we included, performed on patients diagnosed according to these criteria, were unlikely to show severe autonomic impairment. Nevertheless, studies with autopsy-confirmed diagnosis that included also patients misdiagnosed in life as other atypical parkinsonism seem to confirm that severe autonomic failure is not a feature of PSP. Despite this, we believe that it is important to describe autonomic features that may not be severe but still clinically relevant and also may have pathophysiological implications. Having knowledge regarding these possible manifestations also in patients with PSP may be extremely useful for the clinical neurologists.

In conclusion, while PSP patients seem to frequently report visceral symptoms, not always these reflect actual dysautonomia. Based from the few studies that performed objective assessments, the autonomic nervous system seems to be relatively preserved/mildly impaired and at least not as compromised as it happens in MSA.

The autonomic nervous system needs to be studied by appropriate instrumental tests that record responses to different stimuli that are dependent on sympathetic and parasympathetic functions and their reciprocal interactions [83]. Unfortunately these tests are complex and have to be performed in specialized laboratories with technical equipment and dedicated personnel that are available in tertiary referral Centres. Nevertheless they are essential to provide a definite answer and further delineate autonomic involvement in PSP.

## Data Availability

Data sharing not applicable to this article.
